# Development of marine biodiversity database (BISMaL) to enable estimations past habitat conditions for marine life in the northwestern Pacific

**DOI:** 10.1093/database/baad081

**Published:** 2023-11-15

**Authors:** Takashi Hosono, Tomoaki Kitayama, Hideaki Saito, Katsunori Fujikura

**Affiliations:** Global Oceanographic Data Center, Japan Agency for Marine-Earth Science and Technology (JAMSTEC), Showa-machi 3173-25, Kanazawa-ku, Yokohama, Kanagawa 236-0001, Japan; Global Oceanographic Data Center, Japan Agency for Marine-Earth Science and Technology (JAMSTEC), Showa-machi 3173-25, Kanazawa-ku, Yokohama, Kanagawa 236-0001, Japan; Global Oceanographic Data Center, Japan Agency for Marine-Earth Science and Technology (JAMSTEC), Showa-machi 3173-25, Kanazawa-ku, Yokohama, Kanagawa 236-0001, Japan; Marine Biodiversity and Environmental Assessment Research Center, Japan Agency for Marine-Earth Science and Technology (JAMSTEC), Natsushima-cho 2-15, Yokosuka, Kanagawa 237-0061, Japan

## Abstract

Global activities involving the collection of marine biodiversity information have provided a large amount of biological observation records that cover various spatiotemporal areas. To predict biological responses or distribution changes in response to environmental changes by using these observation records, it is essential to analyze not only the current marine physicochemical environmental conditions but also the past conditions when the organisms were observed. We developed a new function to estimate the past marine environmental conditions for the observation records in our marine biodiversity database (Biological Information System for Marine Life: BISMaL) and examine whether the database can reliably estimate thermal habitats for both benthic and planktonic marine organisms. For the benthic squat lobster *Shinkaia crosnieri*, the estimated and observed *in situ* temperatures were similar to each other. For the planktonic chaetognaths *Krohnitta pacifica* and *K. subtilis*, the estimated temperatures showed clear seasonal changes specific to their distribution areas. These results indicated that BISMaL can reliably provide past habitat conditions regardless of planktonic or benthic lifestyles. BISMaL, which provides both biological observations and estimated past environmental conditions through web services, could lower the barrier to data access and use and make data-driven science available not only for data scientists but also for various marine scientists, such as taxonomists, ecologists and field scientists.

**Database URL:**  https://www.godac.jamstec.go.jp/bismal/e/

## Introduction

Global efforts to collect marine biodiversity information, such as Ocean Biodiversity Information System (OBIS, https://obis.org/) and Global Biodiversity Information Facility (https://www.gbif.org/), have compiled a large number of biological observation records that cover various spatiotemporal areas. Based on these records, many studies have been published, including predictions of biological community responses to ocean warming (1–3) and latitudinal patterns of marine biodiversity (4–7). To predict biological responses or distributional changes in response to global environmental changes by using these observation records, it is essential to analyze not only current environmental conditions but also past conditions when organisms were observed. Of the vast number of accumulated observation records, however, biological observation records that have associated environmental measurements such as water temperature or salinity are very limited. To assess this issue, we attempted to develop a new function in a biodiversity database to estimate the associated environmental conditions at the time of past biological observations.

The Biological Information System for Marine Life (BISMaL) is a database that accumulates and disseminates marine biodiversity data derived from scientific research mainly in the northwestern Pacific (8, 9) and was developed and began operating in 2009. Currently, over 1 700 000 biological observation records are published in BISMaL, but a very limited number of the records have associated environmental measurements. Estimating associated environments for biological observation records can be performed by referencing open data that are part of a global reanalyzed ocean dataset such as the World Ocean Atlas (WOA, 10), which provides environmental data with a resolution of 0.25° grids (see https://accession.nodc.noaa.gov/NCEI-WOA18). When available, it is preferable to use a regionally specialized ocean dataset with a higher resolution. In the northwestern Pacific region, Usui *et al.* (11) produced the Four-dimensional Variational Ocean ReAnalysis (FORA) dataset (https://www.godac.jamstec.go.jp/fora/e/index.html) over 33 years with a resolution of 0.1° grids. Such regionally specialized ocean datasets with high data resolution are valuable as a source of information about the past environment. Therefore, we integrated FORA into BISMaL to attempt to regenerate past habitat conditions for biological observation records by referring to the spatially and temporally closest environmental conditions.

In this paper, we introduce the BISMaL database, which integrates ocean datasets to estimate past habitat conditions for marine organisms, and then examine whether BISMaL can estimate reliable habitat conditions for marine organisms. Specifically, the thermal habitats of marine organisms with two different lifestyles (benthic and planktonic species) were estimated: one is a benthic squat lobster, *Shinkaia crosnieri* (Baba and Williams, 1998), that is distributed in deep-sea hydrothermal vents, and the others are two planktonic chaetognaths, *Krohnitta pacifica* (Aida, 1897) and *K. subtilis* (Grassi, 1881), that are commonly observed around Japan. The reliability of the estimated thermal habitat of *S. crosnieri* was examined by comparing estimated values and actual measured values when the species was observed. For the two *Krohnitta* species, whether the estimated thermal habitat can accurately represent seasonality specific to Japanese waters was examined. Additionally, we examined whether a data-driven hypothesis can be extracted by detecting differences between the estimated thermal habitats of the two *Krohnitta* species.

## Materials and methods

### Data in BISMaL

**Figure 1.**
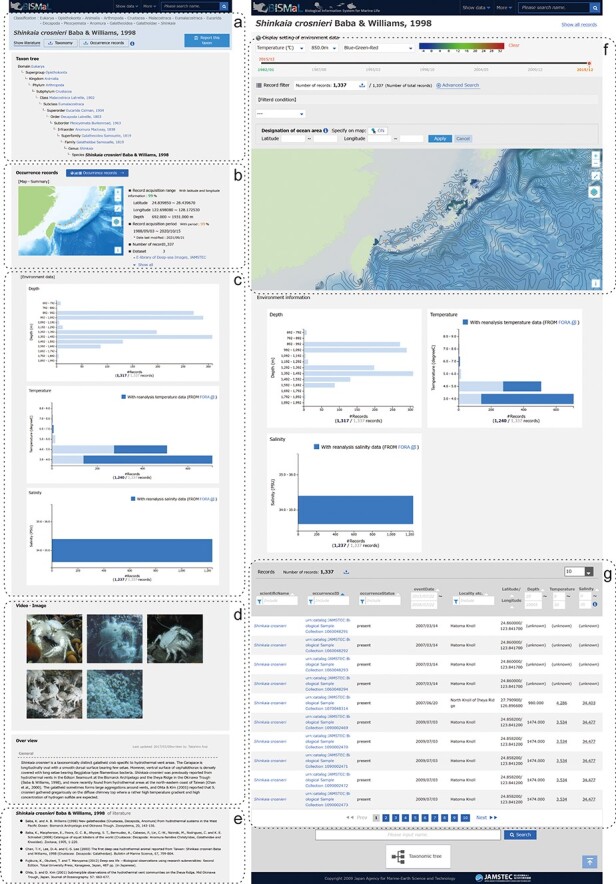
Screenshots of the BISMaL web page for *S. crosnieri*. Left panel: a taxonomic information page (https://www.godac.jamstec.go.jp/bismal/e/view/9000078) composed of taxonomic tree (a), map view of biological observation records (b), histograms of environmental conditions (c), related images and notes (d) and references (e). Right panel: a biological observation record page (https://www.godac.jamstec.go.jp/bismal/e/occurrences?taxon=9000078) composed of map view with estimated environmental contours (f) and observation records (g). Estimated salinity or temperature for observation records is shown as underlined values.

Data in BISMaL are mainly taxonomic information (Figure 1, left panel) and biological observation records (Figure 1, right panel). Observation records contain latitude, longitude, scientific name, date-time, observation methods and other terms in Darwin Core format 2.0, which is an international data standard for exchanging biodiversity information (12). Taxonomic information is a list of scientific names extracted from taxonomic research papers, books or monographs. Each record in the list is composed of scientific name, taxonomic rank, references, accepted/unaccepted situation, Japanese common name, registered date/modified date and national Red List status in Japan. In February 2021, 54 877 scientific names were registered in BISMaL, of which 19 252 taxa had biological observation records. Maintaining the observation records and the taxonomic information within a single database allows flexible handling of the records. For example, in the case of trying to access records for a given scientific name, all records with the scientific name and its unaccepted (synonymized) scientific names can be retrieved without omission by systematically defining the relations between an accepted and unaccepted name. Furthermore, by defining a taxonomic hierarchical structure in the system, observation records of a higher taxonomic rank (e.g. order or family level) can be collectively retrieved from the records of its subordinate rank.

### Ocean physicochemical dataset

To determine the environments at a given point where a biological observation record was obtained, we adopted the ocean dataset FORA, which is a regenerated oceanographic physicochemical condition dataset spanning 33 years in the northwestern Pacific (11). FORA provides daily Network Common Data Form (NetCDF) files from 1 January 1982 to 31 December 2014. Each NetCDF file consists of temperature and salinity data at depths of 0–6300 m with 54 layers and in the range of 117°E–160°W and 15°–65°N with a resolution of 0.1°. In matching biological observation records with FORA environmental data, the latitude, longitude, depths and date of the observation records were adjusted to the data resolution in FORA. Latitude and longitude were rounded down to the nearest 0.1°, and the shallowest depth in the observation record was used as a representative value and then matched to the FORA depth layer containing the representative depth value. Where the date in observation record was given as a period, the earliest date in the period was used as the representative value for matching.

FORA values assigned to observation records are displayed as summarized histograms of water temperature and salinity for each taxon page (Figure 1c) and as underlined values in each biological observation record (Figure 1g). It is also possible to display the distribution of the observation records overlaid on the contour map of temperature or salinity (Figure 1f).

### Database implementation

All data in BISMaL were stored and managed using PostgreSQL (https://www.postgresql.org/). The interface components of the website were designed and implemented using the JavaServer Faces 2.2.20 (https://jakarta.ee/specifications/faces/) and PrimeFaces 10.0.9 (https://www.primefaces.org/). Maps presenting the geographical distribution of biological observation records were drawn by GeoServer 2.20.1 (http://geoserver.org/). The website was successfully tested in several popular web browsers including Google Chrome and Firefox.

Of the data in BISMaL, biological observation records are systematically shared with the OBIS global database. All observation records, which have valid values for eight required Darwin Core terms defined by OBIS, are stored in a system (Integrated Publishing Toolkit) for data sharing (https://www.godac.jamstec.go.jp/ipt/), harvested by OBIS in real time and then integrated into the OBIS database.

### Validation of thermal habitats of benthic species: *S. crosnieri*

We selected the hydrothermal vent squat lobster *S. crosnieri* as a benthic species for validation, because there were sufficient observation records with an actual measured temperature when the species was observed. Observation records of *S. crosnieri* (1254 records) in BISMaL are mainly reported from the dataset of ‘JAMSTEC e-library of deep-sea images’ (J-EDI, 13). The J-EDI dataset archives observation records based on the videos and images taken by deep-sea submersibles and environmental data, including depth, dissolved oxygen, water temperature and salinity that were measured by conductivity-temperature-depth (CTD) attached to the submersibles. The reliability for the estimated thermal habitat of the species was validated by direct comparison with estimated temperatures from FORA and measured *in situ* temperatures by CTD.

### Planktonic species: *K. pacifica* and *K. subtilis*

Because there are sufficient available observation records, which have all latitude, longitude, depth and date information needed to estimate temperature, we selected two chaetognaths, *K. pacifica* and *K. subtilis*, as planktonic species for validation. Observation records of chaetognaths are mainly reported from the ‘JODC Dataset’ (14), which are long-term plankton survey data around Japan from 1951 to 2006. BISMaL archived a total of 904 records of *K. pacifica* and 1136 records of *K. subtilis* during 1982–1992, and there were no *in situ* temperature data for the two species. The two *Krohnitta* species are both categorized as epiplanktonic (>150 m) species (15), but *K. subtilis* has also been reported as mesopelagic in the eastern area of Japan (16) and Sagami Bay (17). As the depth data for all observation records of the two species were reported as ranges (mostly 0–200 m), BISMaL used a depth of 0 m for the estimation. To verify the reliability of the estimated thermal habitat for the two *Krohnitta* species, we estimated experienced temperatures based on the latitude, longitude, depth and date/time of the observation records and examined whether the temperatures could serve to recreate the seasonal changes specific to the sea around Japan. As a similar service to estimate past water temperature, OBIS provides the package *obistools* (Provoost and Bosch, 2019) for R (18), and the *lookup_xy* function in the package returns surface temperatures by referring to the WOA. We compared the results between BISMaL and the *obistools* package.

To detect the differences between the estimated thermal habitats of the two *Krohnitta* species, patterns of estimated temperature compared to latitudinal changes during the high-temperature season (June–October) were visualized using 2D kernel density estimation with the *kde2d* function in the R package *MASS* (19).

## Results

Among a total of 1 713 682 observation records (19 252 taxa) in BISMaL, there were only 25 023 records (1627 taxa) with water temperature measurements (data accessed on 19 February 2021). Estimation of water temperatures was performed for 203 897 records (5778 taxa) by referring to FORA based on date, latitude, longitude and depth.

### Validation of thermal habitats of benthic species: *S. crosnieri*

**Figure 2.**
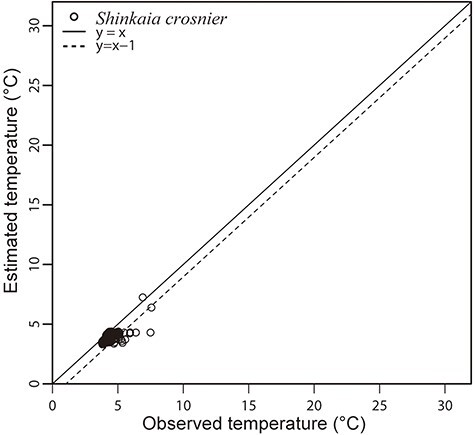
Observed *in situ* temperatures by CTD of *S. crosnieri* habitat and estimated temperatures by BISMaL based on FORA. When the observed *in situ* temperature corresponds to the estimated temperature, data are plotted along the solid line (*y* = *x*).

Among the 1254 records of *S. crosnieri* in BISMaL, 392 records included observed *in situ* temperatures with narrow temperature ranges of 3.7–7.5°C (4.2 ± 0.45, mean ± SD; Figure 2). The estimated values for the 392 records were 3.3–7.2°C (3.8 ± 0.36, mean ± SD), a range similar to that of the observed values. The estimated values were consistently lower than the observed *in situ* values, but the difference between the observed and estimated values was within 1°C (96.1% of the data were plotted between the solid line and the dotted line in Figure 2).

### Validation of thermal habitats of planktonic species: *K. pacifica* and *K. subtilis*

**Figure 3.**
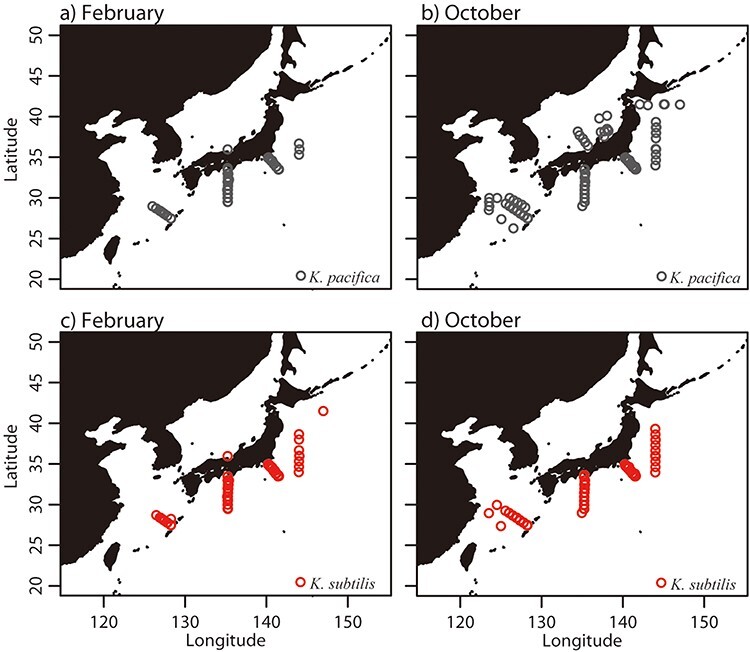
Distribution of biological observation records of *K. pacifica* in February (a) and October (b) and *K. subtilis* in February (c) and October (d). Data are pooled over 11 years (1982–1992).

The occurrence patterns of the two species mostly overlapped throughout the year (Supplementary Figure S1); for example, the occurrence points of the two species overlapped in February (Figure 3a and c). However, in October, the occurrence points of the two species overlapped in the Pacific, while only *K. pacifica* occurred in the Sea of Japan (Figure 3b and d). *K. pacifica* was repeatedly observed in the Sea of Japan during September–October (mostly in October) in 1982, 1983, 1988, 1991 and 1992, while *K. subtilis* was observed only in February 1988.

**Figure 4.**
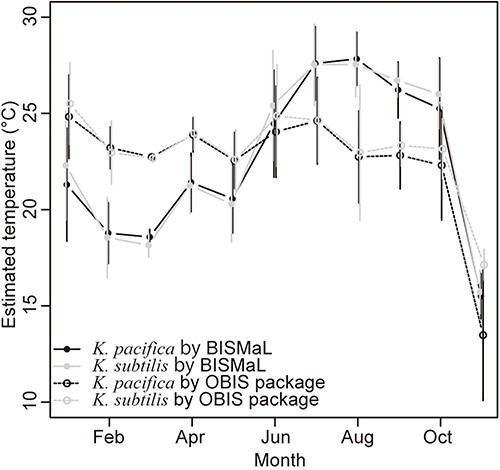
Monthly mean temperatures for the two *Krohnitta* species, which were estimated by BISMaL with FORA (solid lines) and the OBIS package ‘obistools’ (dashed lines) with WOA. Vertical bars indicate standard deviation.

Except for November, which had very limited data, the mean estimated temperatures in BISMaL showed clear seasonal changes in *K. pacifica* and *K. subtilis* (Figure 4), and there was little difference in the monthly mean temperatures between two *Krohnitta* species (monthly mean in *K. pacifica* and *K. subtilis*: 18.8°C and 18.6°C in February and 25.3°C and 26.0°C in October, respectively). On the other hand, seasonal changes in the estimated temperatures from the *obistools* library were small (monthly mean in *K. pacifica* and *K. subtilis*: 23.2°C and 23.0°C in February and 22.3°C and 23.2°C in October, respectively).

**Figure 5.**
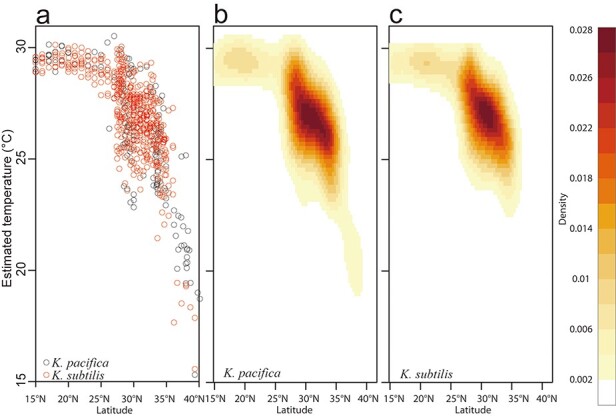
Visualization of estimated thermal habitat across latitude for the two *Krohnitta* species, scatterplot of estimated temperatures (a) and 2D kernel density fitting for *K. pacifica* (b) and *K. subtilis* (c).

The distributions of estimated temperatures across latitude for the two species mostly overlapped (Figure 5a). The kernel density distributions showed almost the same shapes, indicating that the centers of the thermal habitats were located at 25–30°C and 25–30°N (Figure 5b and c). In the areas >35°N and <23.5°C, however, there was a difference in the estimated thermal habitats of the two species, and only *K. pacifica* expanded its range to high-latitudinal and low-temperature areas.

## Discussion

By developing a function referencing past environmental conditions in the BISMaL database, we were able to estimate past habitat conditions for marine organisms regardless of their lifestyle as benthic or planktonic. For the benthic squat lobster *S. crosnieri*, the thermal habitat was estimated to be 3.3–7.2°C, which was similar to the range of observed *in situ* temperatures. For the planktonic species *K. pacifica* and *K. subtilis*, their estimated temperatures showed clear seasonal changes, from 18°C to 26°C from January to October. In addition, visualizing the thermal habitats of the two *Krohnitta* species showed that the distributions of the observed records mostly overlapped but differed in their marginal areas.

The estimated and observed *in situ* temperatures for *S. crosnieri* were similar to each other; however, there was a constant bias in that the estimates were lower than those observed in most cases. The observed temperatures were measured by a CTD attached to a submersible that captured and recorded videos of the species around hydrothermal vents. Therefore, water temperatures near the CTD could have been affected by the hydrothermal activities of the vents. Tsuchida *et al.* (20) measured temperature in a *S. crosnieri* habitat and a hydrothermal vent directly and reported that the temperature at the vent was 301°C and the temperature in the *S. crosnieri* habitat (1–2 m away from the vent) was 4.0–6.2°C. As the reported temperatures in the habitat are not largely different from our estimates (3.3–7.2°C), our result is considered to be reasonable. However, it is notable that the temperature estimation based on BISMaL is preferable for detecting averaged environments in a 0.1° grid, which is a resolution of FORA, and not preferable for detecting unique and specific phenomena such as hydrothermal vents.

For the planktonic chaetognath genus *Krohnitta*, BISMaL estimated seasonal temperature changes of 18–26°C around Japan, which are typical, while the OBIS packages estimated no clear temperature change of 22–23°C. Focusing on the area within 25–35°N and 125–150°E around Japan where a large part of the observed records of the two *Krohnitta* species were obtained, clear seasonal changes in mean surface temperature during 1991–2020 are reported as 18–21°C in February and 27–29°C in August (21, https://www.data.jma.go.jp/gmd/kaiyou/data/db/kaikyo/knowledge/sst.html), and these temperature changes are close to our results. Therefore, BISMaL is able to estimate past thermal habitats reliably and provides an improved estimation over those from existing services, such as the OBIS packages, by using the regional specialized ocean data FORA. However, it is notable that all *Krohnitta* records in this study have a depth range of 0–200 m, which indicates vertical net towing, and BISMaL used the shallowest depth (0 m) for the estimations. Miyamoto *et al.* (17) investigated the vertical distribution of chaetognaths in Sagami Bay and reported that the mean depths were different between the two *Krohnitta* species (30 m and 194 m in *K. pacifica* and *K. subtilis*, respectively). In future, if highly precise depth information for the two species becomes available, then differences in thermal habitat between the two species may be detected more precisely.

Our results showed that the distribution of the two *Krohnitta* species mostly overlapped; however, *K. pacifica* extended its distribution to the Sea of Japan and higher latitude areas in the high-temperature season. Kuroda *et al.* (16) reviewed the fauna, distribution ecology and community structures of pelagic chaetognaths around Japan and reported that the two *Krohnitta* species were observed in all sea areas except the Okhotsk Sea. However, knowledge about the detailed life cycle of the two species is limited around Japan. This may be partially because the two *Krohnitta* species are not conspicuously dominant in every area. Johnson and Terazaki (22) investigated chaetognath species composition in Kuroshio warm-core ring waters, which are eddies of warm water detached from large current systems (see Supplementary Figure S2), and reported that *K. pacifica* was a Kuroshio water indicator species and *K. subtilis* was an offshore water indicator species. It is likely that *K. pacifica*, whose distribution is more affected by Kuroshio waters than that of *K. subtilis*, temporarily appears in the Sea of Japan along the Tsushima Current, which is a branch of the Kuroshio Current (see Supplementary Figure S2). Nagai *et al.* (23) studied the occurrence of chaetognaths, including the two *Krohnitta* species, at a line transect in the Sea of Japan during 1972–2002 and noted that the occurrence of *K. subtilis* was rare in the Sea of Japan, which supported our results. Our results clearly showed the spatiotemporal differences in the marginal habitats of the two *Krohnitta* species. Although the ecological importance of the difference cannot be determined from our data, the difference highlights what needs to be addressed to understand the whole life history of the two *Krohnitta* species around Japan.

In this study, we developed a region-specific marine biodiversity database BISMaL to enable the estimation of physicochemical environmental conditions and showed that it is possible to reliably estimate thermal habitats for marine organisms regardless of their lifestyles. The progress in ocean observation technology will accelerate the accumulation rate of biological observation records in many regions, and smooth integration of the regionally accumulated observation records in a global level will enhance the environment for data-driven researches in marine biodiversity. In fact, BISMaL has achieved smooth data sharing with the OBIS global database, and the data from BISMaL contributes as data to interpolate the Northwest Pacific region in enhancing environment for global marine biodiversity studies. However, it is important to mention that the enhancement of these data-driven research environments is not only for pure data scientists. BISMaL provides an easily accessible platform of both biological and environmental data as a web service, and this could open the door to data-driven science for not only pure data scientists but also for a variety of marine researchers, such as taxonomists, ecologists and field investigators. This approach could broaden the limit and encourage greater engagement with research communities outside specific areas of scientific research.

## Supplementary Material

baad081_Supp

## Data Availability

The data underlying this article are available in BISMaL database at https://www.godac.jamstec.go.jp/bismal/ and can be freely accessed.
