# Supplementary figures and images for: Development of marine biodiversity database (BISMaL) to enable estimations past habitat conditions for marine life in the northwestern Pacific
Source: Database (Oxford). 2023 Nov 15;2023:baad081. doi: 10.1093/database/baad081 (PMC10952404; doi:10.1093/database/baad081)

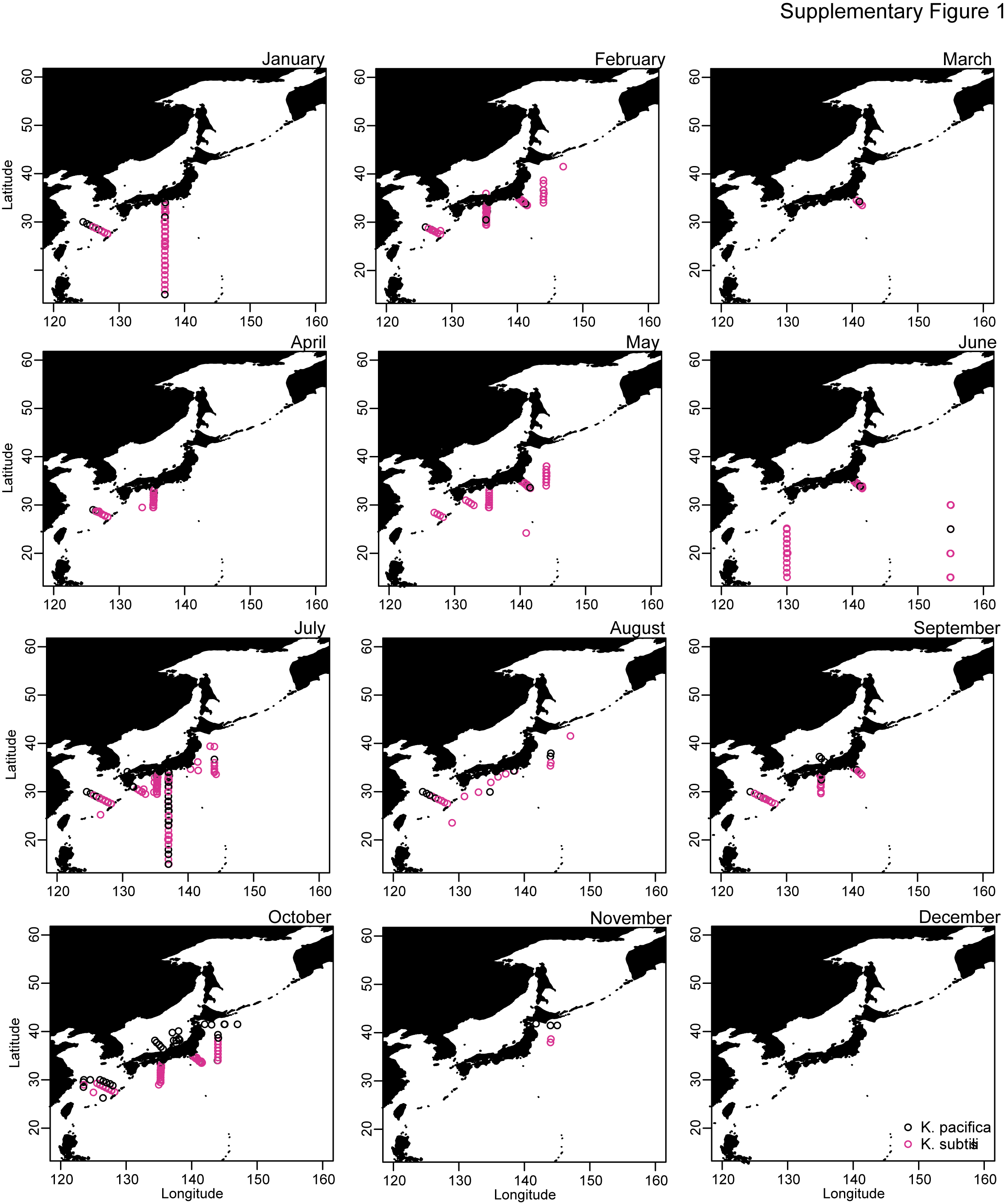

Supplement: baad081_Supp [file baad081_supp.zip › suppl_data/SF1.jpg]

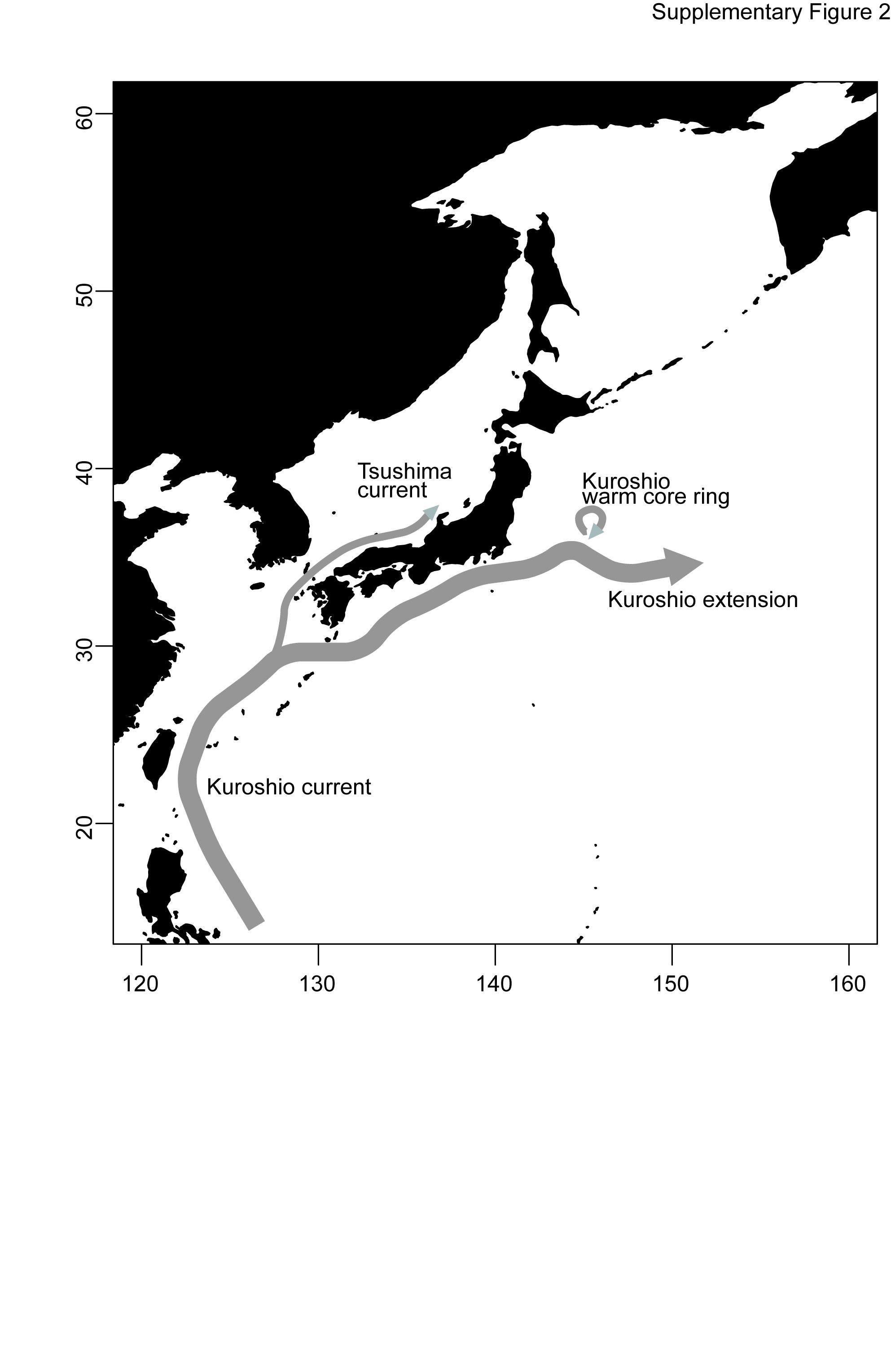

Supplement: baad081_Supp [file baad081_supp.zip › suppl_data/SF2.jpg]
